# Computational Discovery of TTF Molecules with Deep Generative Models

**DOI:** 10.3389/fchem.2021.800133

**Published:** 2021-12-23

**Authors:** Alexander Yakubovich, Alexey Odinokov, Sergey Nikolenko, Yongsik Jung, Hyeonho Choi

**Affiliations:** ^1^ Samsung R&D Institute Russia (SRR), Samsung Electronics, Moscow, Russia; ^2^ Steklov Institute of Mathematics at Saint Petersburg, Saint Petersburg, Russia; ^3^ ISP RAS Research Center for Trusted Artificial Intelligence, Moscow, Russia; ^4^ Samsung Advanced Institute of Technology (SAIT), Samsung Electronics, Yeongtong-gu, South Korea

**Keywords:** generative model, OLED, organic light emitting devices/display, computational materials discovery, quantum chemistry, autoencoder, molecular database screening

## Abstract

We present a computational workflow based on quantum chemical calculations and generative models based on deep neural networks for the discovery of novel materials. We apply the developed workflow to search for molecules suitable for the fusion of triplet-triplet excitations (triplet-triplet fusion, TTF) in blue OLED devices. By applying generative machine learning models, we have been able to pinpoint the most promising regions of the chemical space for further exploration. Another neural network based on graph convolutions was trained to predict excitation energies; with this network, we estimate the alignment of energy levels and filter molecules before running time-consuming quantum chemical calculations. We present a comprehensive computational evaluation of several generative models, choosing a modification of the Junction Tree VAE (JT-VAE) as the best one in this application. The proposed approach can be useful for computer-aided design of materials with energy level alignment favorable for efficient energy transfer, triplet harvesting, and exciton fusion processes, which are crucial for the development of the next generation OLED materials.

## 1 Introduction

Operation of organic light emitting and photovoltaic devices can be greatly improved by utilizing the triplet–triplet fusion (TTF) process, when two triplet excitons of low energy merge into one singlet exciton of higher energy (Gray et al., 2014). Despite some successes in the discovery of TTF materials (Kondakov, 2015), their number is still limited, the main reason being strict requirements on the alignment of the lowest singlet and triplet energy levels that is difficult to satisfy by randomly picking a compound (Gómez-Bombarelli et al., 2016; Wang et al., 2020).

**FIGURE 1:**
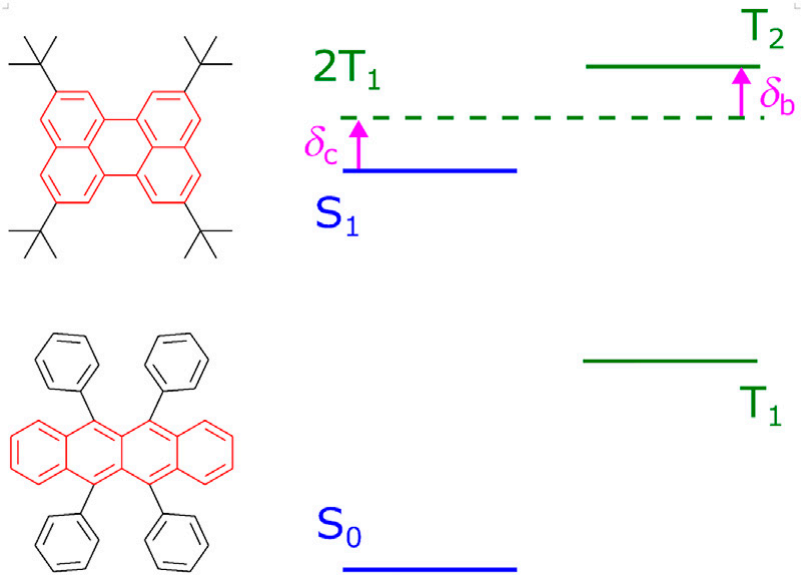
Typical TTF materials, tetra-tert-butylperylene (Ravetz et al., 2019) and rubrene (Cheng et al., 2010), and the scheme of energy levels favorable for the TTF process. Cores of the compounds are highlighted in red. For efficient TTF, energy differences *δ*_*b*_ and *δ*_*c*_ should be positive.

Compounds with TTF activity often contain a “core”, a fused heterocyclic fragment responsible for their basic properties, as shown in Figure 1; then redox potentials and excitation energies of the compound can be further modified by adding side groups. A convenient way of designing new materials is to start from an already known prototype and modulate its properties by varying functional groups. Moreover, any core requires certain chemical modifications to become a real-life TTF material due to technology-related reasons; these modifications may be needed to increase solubility, prevent undesired aggregation, or reduce photochemical degradation. In such cases, one should be careful not to spoil a promising core by inappropriate substitutions. Moreover, another strict constraint appears in the case of deep blue OLED emitters, namely high singlet excitation energy *S*_1_, which makes it extremely difficult to perform concise chemical modifications. Under these circumstances, it becomes especially important to find new original cores with favorable arrangements of energy levels. An efficient search strategy should be able to sample the space of functionalized heterocyclic compounds and suggest candidates based both on the core structure and nature of the side groups.

In this work, we demonstrate a general and computationally efficient approach for the search for novel TTF materials. The approach is based on three steps. First, we generate all possible polycyclic molecular graphs within predefined limits and then decorate them with heteroatoms and side groups, allowing for dense coverage of large regions in the chemical space. Second, we apply a fast semiempirical (SE) method to calculate low-lying singlet and triplet energy levels, allowing for high-throughput screening of molecular databases. Third, we use a generative machine learning (ML) model based on deep neural networks to suggest new compounds with the distribution of generated molecules biased towards blue TTF emitters. In particular, we compare several different generative models and choose the best one for further discovery of leads in a larger chemical space.

Modification of cores with side groups greatly expands the considered areas of chemical space and makes it necessary to apply ML-based models to perform guided search for promising candidates. For a comprehensive validation of ML-based models, we have conducted a complete screening of a subset of the relevant “core” chemical space, which has allowed us to choose the best deep generative model for the task. Design of novel TTF materials is based on a multi-step workflow that begins with the generation of training datasets and repeatedly provides lead compounds in a batch-wise manner, aiming to provide leads for further expert-based selection and experimental trials.

## 2 Methods

### 2.1 Target Properties of TTF Candidates

The triplet-triplet fusion process occurs when two *T*_1_ excitations transform into one excited singlet state. To ensure high internal conversion efficiency, it is important to suppress the formation of higher triplet states. Therefore, a criterion for a molecule to be an appropriate candidate for the design of a TTF material is usually expressed in terms of the lowest singlet (*S*_1_) and two lowest triplet (*T*_1_ and *T*_2_) energy levels as follows:

$$2T_{1}>S_{1},\;2T_{1}<T_{2},$$ (1)

where the first inequality ensures that there is enough energy in two triplet states to form a singlet excitation, and the second inequality prohibits the formation of higher excited triplet states, thus favoring only singlet excitation formation. Large splitting between *S*_1_ and *T*_1_ implies that both states originate from ππ* excitations. Under this assumption, it makes sense to focus the methodology on accurate prediction of ππ* states, and tolerate lower performance for nπ* states. For example, ππ* excitations are relatively unaffected by the solvent polarity, so vacuum calculations should be sufficient and allow for faster computations.

The present work is focused on blue OLED light-emitting materials that require a certain threshold for *S*_1_ energy. In particular, we can define three numerical criteria to filter compounds appropriate for TTF applications as follows:

$$\begin{array}{ccc} S_{1} & > & \delta_{a},\\ T_{2}-2T_{1} & > & \delta_{b},\\ 2T_{1}-S_{1} & > & \delta_{c}, \end{array}$$ (2)

where numerical values of the threshold parameters *δ*_*a*_, *δ*_*b*_, and *δ*_*c*_ can be adjusted to find a better tradeoff between the number and quality of final candidates. In the ideal case, we should set *δ*_*a*_ = 2.8 eV, *δ*_*b*_ = *δ*_*c*_ = 0 eV, but in practice we use less restrictive values to allow for intrinsic inaccuracies of simulation approaches and finite width of excitation energy levels of the molecules in the OLED emission layer.

### 2.2 Algorithm for the Generation of Molecular Topology

**FIGURE 2:**
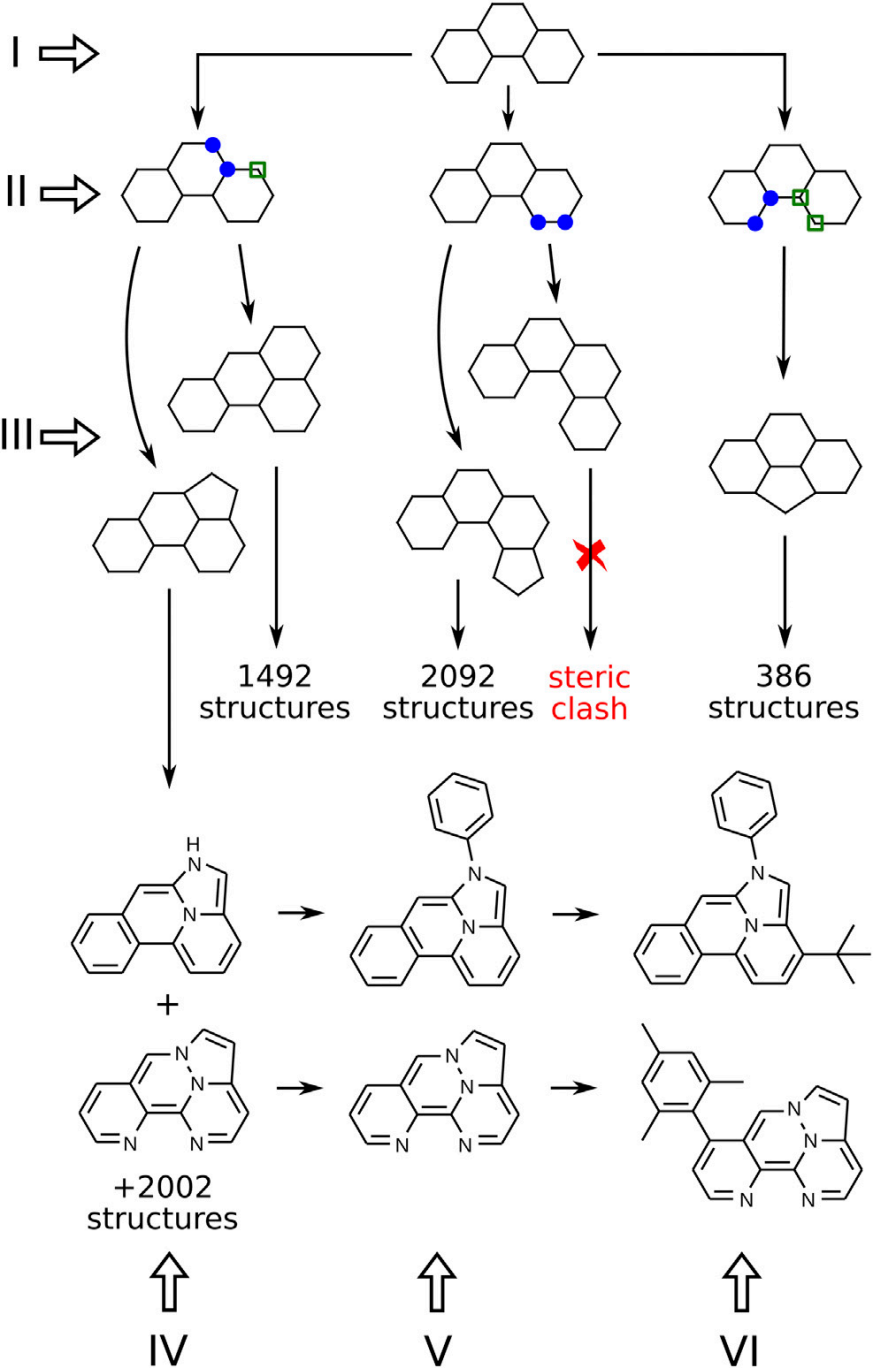
Algorithm for the generation of molecular structures. Numbers show the actual number of descendants that can be obtained from a given structure (but are not shown in the figure). Blue markers indicate two neighboring sites chosen for ring fusion. Sites with green markers are added automatically to the new ring.

In this section, we present our algorithm for the generation of molecular structures. It includes several consecutive steps, illustrated in Figure 2 with Roman numerals. The procedure can be subdivided into two parts. First, we generate a skeleton frame, that is, a graph of connected points, that does not yet specify the atomic types or bond orders (steps I-III in Figure 2). Second, the frame needs to be populated with heteroatoms, double bonds, and side groups that correspond to the correct Kekulé structure of a specific molecule (steps IV-VI in Figure 2).

Figure 2 presents a branch of the structure generation tree that starts from a single frame made of three 6-membered rings (step I). On step II we need to mark the places where a new ring can be attached. All possible pairs of connected atoms located on the perimeter of the frame should be considered. On step III, 5 and 6-membered rings are attached to the marked places. Duplicates already stored in the database are removed, and some simple heuristics are applied to filter out structures with steric hindrances. After processing all frames consisting of *N*-membered rings, we obtain the next “generation” of frames consisting of (*N* + 1)-membered rings.

On step IV, heteroatoms are placed in the frame according to the following rules: at most 4 heteroatoms in the frame; only nitrogen and oxygen are considered; even number of *π* electrons is required; no pair of heteroatoms can be connected by a covalent bond except for two nitrogens. The last rule is introduced to exclude extremely exotic compounds with peculiar distributions of heteroatoms in the molecule that *a priori* have a very little chance to be synthesizable and photostable.

If the resulting labeled graph can be successfully kekulized with *openbabel* (O’Boyle et al., 2011), it is considered to be a valid molecule, and its SMILES string is stored in the database. Again, all duplicates are removed. The resulting molecules comprise the set of “cores”, compact polycyclic fragments without side groups that can serve as building blocks to make more complex TTF materials. The chemical space of cores made of 4 or less rings consists of 472, 505 structures, and all of them can be enumerated with the above algorithm.

To make the resulting materials more likely to be applicable in real-life OLEDs, the cores should be modified further. First of all, the N-H bond in secondary amines should be capped with some residue because of low photochemical stability of the N-H bond. We replaced hydrogens with phenyls to simulate adjacent (presumably aromatic) parts of the complex TTF material (see step V in Figure 2). On step VI, the required number of side groups can be placed at the given positions, leading to the final compound.

### 2.3 Experimental Data for Validation

We have collected data from the literature on well-resolved 0–0 transitions of 55 polycyclic molecules with measured absorption or luminescence from the first singlet or triplet excited states (Halverson and Hirt, 1949; Sponer and Rush, 1949; Halverson and Hirt, 1951; Hirt et al., 1954; Evans, 1957; Ito et al., 1957; Goodman and Kasha, 1958; Goodman, 1961; Shimada, 1961; Dorr and Gropper, 1963; Gropper and Dorr, 1963; Burgos et al., 1977; Schmidt, 1977; Schiedt and Weinkauf, 1997; Reineke and Baldo, 2014; Padula et al., 2019). The structures are presented in Supplementary Figure S2. The compounds can be clearly divided into three groups: pure aromatic hydrocarbons (composition HC), nitrogen-containing compounds (composition HCN) and oxygen-containing compounds (composition HCNO). We made no distinction between absorption and luminescence, since we used data on 0–0 transitions. We also used experiments performed in different media: gas phase, non-polar solvents, rigid matrix or alcohols. Water and other highly polar solvents were not present. We compare this experimental data with calculations performed in the vacuum; this is a reasonable simplification due to the weak dependence of ππ* transitions on the solvent polarity. In any case, data points obtained in different solvents follow the same trend, and the number of experiments performed in every particular medium is too low for reliable statistical analysis. Under these approximations, we were able to collect a dataset suitable for the validation of the utilized computational approach.

### 2.4 Calculation of the Excitation Energies

Meeting growing needs of computational chemistry, various benchmark molecular datasets are being continuously created nowadays (Wu et al., 2018). A typical dataset contains molecular structures and properties calculated using density functional theory (DFT). One of the most famous developments of this kind in the area of material science has been the Harvard Clean Energy Project (Hachmann et al., 2011), spanning 2.3 million candidate organic photovoltaic materials. However, most datasets do not provide spectral properties since the calculation of excited states using time-dependent DFT (TDDFT) is more time-consuming and often less reliable than the calculation of the ground state. On the other hand, datasets containing spectral properties are either not large enough (Abreha et al., 2019) or have small overlap with compounds relevant for TTF applications (Wu et al., 2018). This makes it necessary to prepare our own training dataset in order to search for candidate TTF compounds.

Since our generated structures amount to four hundred thousands compounds with more than 10 heavy atoms in average, the use of TDDFT to assess spectral properties is extremely computationally expensive. We estimate that TDDFT computations for a dataset of 0.5 million TTF molecules would require more than 100 CPU-years. Moreover, the validity of TDDFT as the correct *ab initio* method is questionable. One well-known issue is, for instance, the uneven treatment of excitations of different nature or spin multiplicity (Parac and Grimme, 2003). Even valence ππ* excitations of polycyclic compounds can pose substantial challenges (Grimme and Parac, 2003; Prlj et al., 2016). In order to combine computational efficiency with accurate prediction of spectral properties, we used semiempirical methods of quantum chemistry. Despite them not being *ab initio* approaches, many semiempirical methods, including “spectral” modifications, were initially parametrized on small aromatic and other flat conjugated organic molecules. The accuracy of semiempirical methods for the prediction of the lowest excitation energies is expected to be on par with TDDFT, while greatly speeding up calculations. One can compare different approaches and estimate their typical errors by validating computational approaches against experimental data. Reference data for a small validation dataset can also be obtained with high-level *ab initio* methods. For molecules of moderate size, such as the TTF cores we consider in this work, even multiconfiguration calculations can be theoretically feasible. We have attempted to apply the complete active space self-consisting field (CASSCF) method supplemented with multiconfiguration second-order perturbation theory (MCQDPT). The maximum reasonable size of active space was (12, 12), which was found to be sufficient for triplet excitation to converge in almost all cases. Unfortunately, first singlet excitations converged much more slowly. Even after some admixture of the ground state, convergence was not achieved. It appears that the CASSCF/MCQDPT approach cannot be used for blind screening without manual inspection of every particular case, so we limited the validation to experimental references.

The first step in the calculation of excitation energies is the optimization of molecular geometry. For this purpose, we have used the SE method PM3 as implemented in the Gaussian 16 software package (Frisch et al., 2016). We used the configuration interaction singles (CIS) approach to compute excitation energies. We have tested three semiempirical methods: AM1 (Dewar et al., 1985), PM3 (Stewart, 1989), and ZINDO/S (Ridley and Zerner, 1973) as implemented in the Gaussian 16 software package. For comparison, we also calculated excitation energies using DFT with the settings optimized for mixed-valence organic compounds (Renz et al., 2009): BLYP35/def2-TZVP (Weigend and Ahlrichs, 2005) geometry optimization followed by TDDFT for *S*_1_ and *T*_2_ states, or by ΔSCF for *T*_1_ state using M062X exchange-correlation functional (Zhao and Truhlar, 2008) and the same basis set. It important to note here that our final goal is to develop a method to predict transition energies within a series of polycyclic organic molecules, so we are not interested in the absolute accuracy of the method but rather in its high precision. Bias can be corrected with a linear transformation applied after the calculation. The performances of different methods are compared in Table 1. From the perspective of these results, we can suggest the SE method PM3 as the optimal choice for all further calculations on large molecular datasets. After proper linear correction, it outperforms other SE methods for triplets and is almost on par with ZINDO/S for singlets. PM3 is also on par with corrected DFT and significantly improves over DFT without correction. Plots of calculated versus experimental transition energies for PM3 and DFT can be found in Supplementary Figure S3.

**TABLE 1:** Root mean squared error (RMSE, in eV) between the predicted and experimental excited state energies. Values for singlet and triplet states are presented separately.

Method	State	RMSE	RMSE,corrected
PM3	S_1_	0.286	0.184
	T_1_	0.720	0.279
AM1	S_1_	0.257	0.233
	T_1_	0.751	0.342
ZINDO/S	S_1_	0.276	0.267
	T_1_	0.835	0.381
DFT	S_1_	0.549	0.173
	T_1_	0.858	0.242

### 2.5 Machine Learning-Assisted Design

Although exhaustive enumeration of chemical compounds is possible for certain restricted areas of the chemical space, it is always desirable to “soften” the constraints and search for promising compounds within less restricted regions. Moreover, it is often not easy to formulate clear and complete rules on the chemical diversity of all possible candidate compounds and implement the corresponding deterministic algorithms for library generation. In such cases, approaches based on machine learning and, in particular, deep neural networks can be of great help. The general idea of ML-assisted design proceeds as follows: first, we construct computationally (or extract from experiments) a database with a certain set of molecules that we assume to be relevant for the considered problem. Then, we train a generative machine learning model (usually a deep neural network) on that database, in the hope that the model will capture fundamental structural and chemical features of the dataset and will be capable of suggesting new molecules beyond the training set. If the architecture of the generative model and learning procedures are organized well, one can expect that a large fraction of generated molecules will be relevant for the problem of interest, thus greatly reducing the search space for subsequent validation. In the particular case of TTF compounds, we expect that the model will generate chemical structures featuring excitation energies applicable for the TTF process (see formula (2) and discussion below for details).

To test the applicability of generative models for computational discovery of TTF materials, we have utilized and compared several deep generative models, including character-level recurrent neural networks, adversarial autoencoders, and variational autoencoders (see Section 3.2). The best model that we recommend for practical use is the junction tree variational autoencoder (JT-VAE) architecture introduced by Jin et al. (2018). The decoder of JT-VAE consists of two parts: a graph convolutional neural network (CNN) and a junction tree convolutional neural network. The choice of the architecture was motivated by JT-VAE’s superior ability to encode and decode cyclic fragments of molecules. The latter is often challenging for conventional molecular graph CNNs but is of primary importance for TTF molecules that feature distributed *π*—electronic orbitals.

### 2.6 Prediction of Excitation Energies

**FIGURE 3:**
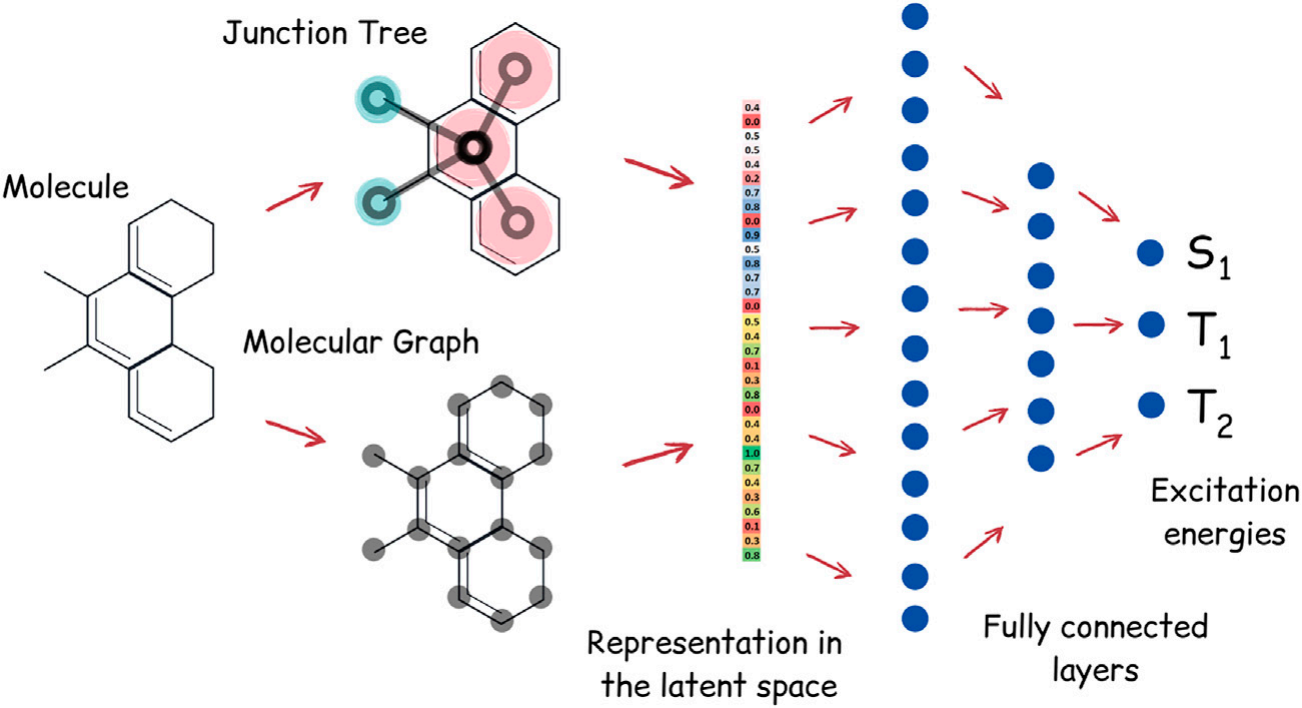
Architecture of the JT-E network for excitation energies prediction. Latent vectors of the JT-VAE encoder corresponding to the junction tree and molecular graph are connected to several fully connected layers. The last layer has three heads, corresponding to *S*_1_, *T*_1_, and *T*_2_ excitation energies.

Apart from the task of molecular generation, neural networks can also be used to predict excitation energy levels of the molecule. We have trained the neural network to predict energies of the singlet and first two triplet states of TTF molecules. Our neural network for energy prediction is also based on junction tree convolutions; the high-level architecture of this neural network, which we call JT-E (Junction Tree Energies), is presented in Figure 3. JT-E is constructed as follows: the layer of Junction-Tree encoder preceding the latent space is connected to a network with several fully connected layers of decreasing dimensionality. The last layer of that network has three heads that correspond to *S*_1_, *T*_1_, and *T*_2_ excitation energies. The neural network is trained to minimize the sum of root mean square errors between predicted and calculated values of excitation energies. The JT-E model has allowed us to predict with good accuracy if a molecule might be suitable for TTF knowing only its SMILES notation (see Supplementary Table S1 for numerical results on existing benchmarks).

## 3 Results

### 3.1 Brute-Force Screening of the Core Compounds

The structure generation algorithm shown in Figure 2 has provided us with 472, 505 non-equivalent compounds with at most 4 rings, which constitutes an exhaustive sampling of the chemical space defined by the constraints listed in Section. 2.2. Applying formula (2), we have selected 5,690 candidates from the set of 472, 505 compounds that are most promising for deep blue TTF applications. These candidates should be subjected to more detailed analysis. An important additional target here is potential synthesizability. The most robust way to ensure synthesizability is to search for already known compounds. Among 5,690 candidates, we found 107 compounds contained in the PubChem (Kim et al., 2021) database. This estimate gives hope that the exploration of considered regions of chemical space can provide a sufficient amount of TTF candidates, both core structures and their derivatives.

Among 107 PubChem hits, several distinct groups of compounds can be identified. The first group of 16 compounds consists of anthracene and its nitrogen-containing structural and isoelectronic analogues. Then, one can found 19 analogues of tetracene, 11 analogues of isobenzofurane, as well as pyrene and two of its analogues. A large and diverse group of 28 compounds contains furane moiety as part of the system of fused rings. The remaining 30 compounds are not so closely related to existing TTF materials. The major part of molecules from the set with 107 elements are analogues of molecules with registered TTF activity (Wang et al., 2020). Introduction of additional nitrogens does not change the electronic configuration, but modulates nuclear charges of chosen atomic sites, which sometimes can make *S*_1_ levels higher (note the cases of tetracene and isobenzofurane), so the conditions for blue TTF materials are satisfied. Review of the core compounds produced by the screening procedure supports the conclusion about the adequacy of applied methodology and underlying SE approach. This success can be partially explained by the loose criteria used in the screening: more than 1% of the original dataset has passed the filters. This is in line with the general strategy of filtering out definitely bad compounds and allowing all that have a chance to prove useful. We believe that the list of PubChem hits contains some indications useful in the search for novel cores with TTF activity. In the subsequent sections, we apply the same computational procedure to prepare training datasets for targeted design of TTF materials based on substituted compounds. The list of PubChem hits, as well as specific details of the screening procedure, can be found in the Supplementary Material (see Note S2.2 and Supplementary Table S2).

### 3.2 Baselines and Performance of Generative Models

We have investigated the performance of various generative models on a dataset of cores defined in the previous subsection. Well-defined chemical composition of the subspace allows us to measure consistently if generative models are capable of suggesting molecules predominantly from the chemical subspace of interest and whether it is possible to tune generators to suggest novel molecules from the subspace with energies satisfying TTF criteria. We have excluded molecules with low and negative excitation energies from the dataset using the following criteria:

$$S_{1}>1.0\;eV,\;T_{1}>0.5\;eV,\;T_{2}>1.0\;eV.$$ (3)

Negative excitation energies are nonphysical and correspond to situations where methods of quantum chemistry fail for low lying excitations. Since we focus on the discovery of TTF molecules suitable for blue OLED applications, we are not interested in those molecules because they will only introduce additional noise to the models. The total number of molecules in the truncated dataset is 341, 433.

**FIGURE 4:**
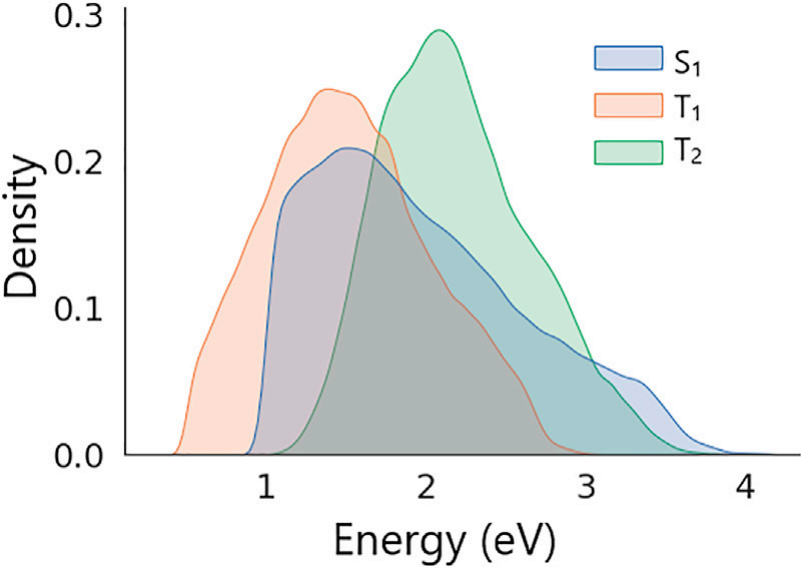
Distributions of *S*_1_, *T*_1_, and *T*_2_ excitation energies in the dataset of generated molecular structures.

Distributions of excitation energies in the dataset are shown in Figure 4. The figure shows that all energies in the dataset feature unimodal distributions with the following means and standard deviations: *S*_1_ = 1.98 ± 0.66 eV, *T*_1_ = 1.52 ± 0.51 eV, and *T*_2_ = 2.19 ± 0.47 eV. The dataset was split randomly into two parts of the same size that were used as training and validation sets (see SI for details).

We have investigated the performance of our implementation of the JT-VAE model (modified from https://github.com/wengong-jin/icml18-jtnn) and three well-known baseline models, namely:

• character-level recurrent neural network (CharRNN) (Preuer et al., 2018; Segler et al., 2018) that models the distribution of the next token in a sequence (SMILES string) with a recurrent neural network; • variational autoencoder (VAE) (Kingma and Welling, 2014; Kadurin et al., 2017; Gómez-Bombarelli et al., 2018)it consists of two networks, encoder and decoder, that learn a mapping of the input into a low-dimensional latent space by minimizing the reconstruction loss and regularization in the form of the Kullback–Leibler divergence between the approximation and the posterior distribution; • adversarial autoencoder (AAE) (Makhzani et al., 2016) that replaces the Kullback-Leibler divergence from VAE with an adversarial objective, training a discriminator network to distinguish samples from the latent space and a prior distribution that the model will sample from to generate new instances.

In all generative models, we use SMILES strings as the input and output representations. We have used the implementations of CharRNN, VAE, and AAE models available athttps://github.com/molecularsets/moses, the benchmarking platform called MOSES (Polykovskiy et al., 2020). All models were trained on the training dataset using hyperparameters and protocols as suggested by Polykovskiy et al. (2020).

We have implemented two different regimes for sampling from the latent space of autoencoder models (VAE, AAE, and JT-VAE): random and seeded. The random regime corresponds to “conventional” sampling of the latent space from the normal distribution N(0,1) that was used as prior in our models. Seeded sampling was carried out as follows. After training the encoder on the training dataset, a subset of molecules most promising for deep blue applications was selected using the criteria from formula (2): *δ*_*a*_ > 2.7 eV, *δ*_*b*_ = *δ*_*c*_ >—0.1 eV. Only 58 out of 171, 716 molecules in the training set satisfy these criteria; we will further refer to molecules satisfying them as *leads*. Latent representation vectors *ν*_*i*_ were calculated for each of the leads. Then, three lead vectors *ν*_1_, *ν*_2_, and *ν*_3_ were selected randomly and multiplied by random positive factors *α*_1_, *α*_1_ and *α*_3_ that satisfy the following relation: $\sum_{i=1}^{3}\alpha_{i}=1$ . The sample vector *ν* in the latent space was constructed as $\nu =\sum_{i=1}^{3}\alpha_{i}v_{i}$ . The molecular structure was obtained by applying the model’s decoder to the resulting latent vector *ν*. This approach has allowed us to sample the latent space not randomly but mostly in the vicinity of known leads. This should be beneficial if the latent space clusters favorably, separating promising TTF molecules from the rest (see discussion in Section 3.3). We have modified the implementations of VAE and AAE models by Polykovskiy et al. (2020) to run sampling in seeded mode. Note that since *CharRNN* is not an autoencoder model, it cannot be “seeded” with leads. Therefore, we do not present any results for *CharRNN* in the seeded mode.

Results for random and seeded sampling of the latent space for different models are presented in Table 2; we have obtained 10, 000 samples from each model. Table 2 clearly shows that all models were capable of suggesting valid SMILES, as checked with RDKit (Landrum, 2012).

**TABLE 2:** Performance of deep generative models for 10, 000 random samples: number of valid molecules, number of unique molecules, number of molecules from the training set, number of molecules from the validation set, number of leads [defined as *δ*_*a*_ > 2.7 eV, *δ*_*b*_ = *δ*_*c*_ >—0.1 eV in formula (2)] from the train and validation sets and their percentage among the corresponding generated samples.

Model	Sampling	Valid	Unique	From train	From valid	Train Leads	% Train Leads	Valid Leads	% Valid Leads
CharRNN	random	9,962	**9,760**	**7,487**	1886	2	0.026	1	0.053
VAE	random	6,312	6,246	1999	1,394	1	0.050	0	0.000
AAE	random	7,582	7,400	2,371	1922	1	0.052	0	0.000
JT-VAE	random	**10 ,000**	9,186	1,026	1,036	1	0.097	0	0.000
VAE	seeded	5,918	5,781	1,617	1,198	7	0.433	1	0.083
AAE	seeded	7,974	7,376	2,643	**2,155**	3	0.114	4	0.186
JT-VAE	seeded	**10 ,000**	3,472	559	558	**16**	**2.862**	**11**	**1.971**

Largest entries in each column are presented with bold font.

One of the most important properties of a generative model is their ability to sample from novel yet not arbitrary regions of chemical space. Since in the present work we are interested in regions of chemical space consisting solely of *π*-conjugated systems with 5 or 6-membered rings, we measure the extrapolating ability of generative models by the number of suggested molecules from the validation set. Note that all generated molecules should not necessarily satisfy the constraints applied during dataset creation, since no formal restrictions on molecular composition were implemented for the generators. Some generated molecules do not belong to the above-mentioned chemical space of *π*-conjugated molecules. Therefore, the number of unique molecules is larger than the sum of molecules from the training and validation sets. Table 2 shows that the AAE model was able to suggest the largest fraction of novel molecules (1992 and 2,155 for random and seeded implementations respectively). Almost 15% of the molecules were from the validation set, i.e., novel. Note also that the size and chemical composition of the training and validation sets are identical, so an unbiased generator should suggest a similar number of molecules from both sets. Autoencoder models indeed demonstrate nearly equal number of generated molecules from both datasets. On the contrary, the *CharRNN* model is extremely biased towards the training set, which could be an indication of overfitting; here we do not investigate that question in depth since we used default suggested values of training parameters from MOSES (Polykovskiy et al., 2020).

The most interesting and important part for the problem of the discovery of novel TTF molecules is the number of novel discovered leads. Recall that a lead is a molecule with excitation energies suitable for blue OLED applications. We see that all models performed poorly in the conventional random sampling mode: *CharRNN* suggested one lead from the validations set, while all other models suggested none. The situation is very different for seeded generation. The AAE model was capable of suggesting four leads from the validation set that have not been seen by the model during training. And this is exactly where the JT-VAE model shines: it was able to generate 11 TTF candidates from the validation set, much higher than any other model in the comparison.

Note also that the number of unique molecules generated by AAE and VAE is nearly identical to the total number of valid SMILES both in random and seeded implementations. For JT-VAE, this holds under random sampling, but not in the seeded mode, where nearly 65% of generated molecules turned out to be duplicates. At first glance it might seem to be a drawback, but in fact this property means that fewer molecules need to be checked for excitation energies favorable for TTF, and it shows that the sampling space of JT-VAE in seeded mode is much more concentrated. Ultimately, we are interested in the number of suggested leads, not just the number of unique molecules, and indeed, despite lower number of unique SMILES, the JT-VAE model suggested by far the most leads from the training and validation sets. This advantage is especially striking if we consider the fraction of generated molecules that need to be checked to find a new lead (shown as percentages in Table 2): the probability to find a lead with JT-VAE is ≈2.4 % for each new suggested SMILES string, which represents a more than 15 times higher rate than for the VAE and AAE models and 75 times higher than picking molecules from the validation set at random.

Note that for all autoencoder models we have observed a presumably linear dependence between the number of unique molecules and the number of molecules from the training and validation sets, as indicated by comparing the random and seeded generators. This is indicative of the fact that seeded generation does not alter the fraction of generated molecules belonging to the desired region of the chemical space, in our case *π*-conjugated systems with 4 rings. This observation allows to suggest that application of seeded sampling does not disturb the predefined constraints on molecular composition, but allows to further accelerate lead discovery.

Based on the above analysis, we suggest the JT-VAE model with seeded sampling as the best generative model for the discovery of realistic candidates for deep blue OLED applications.

### 3.3 Structure of the Latent Space

**FIGURE 5:**
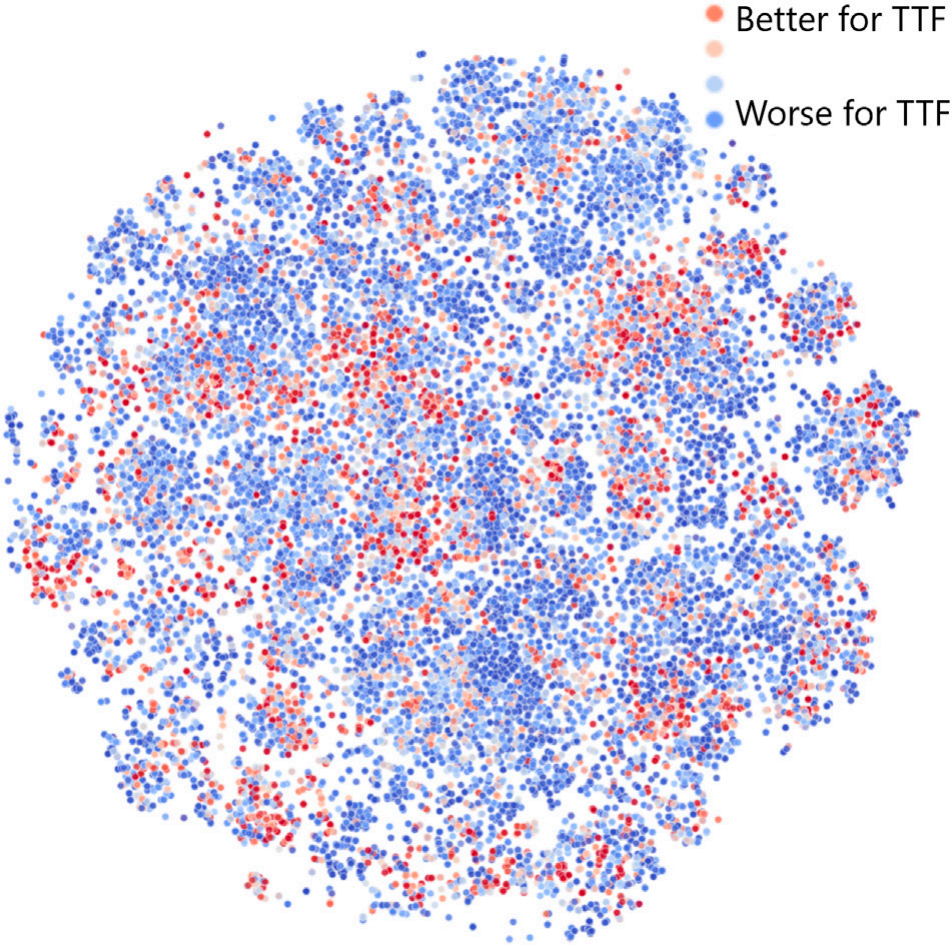
Structure of the JT-VAE latent space model obtained by dimensionality reduction via t-SNE (van der Maaten and Hinton, 2008). Color corresponds to the “fitness” of a molecule for TTF applications: red denotes a better fit, blue, a worse fit.

To better understand why and how the JT-VAE model generates an increased number of leads with seeded sampling, we have investigated the latent space of the model using the t-distributed Stochastic Neighbor Embedding (t-SNE) to generate a two-dimensional visual representation (van der Maaten and Hinton, 2008). The results obtained for 25, 000 molecules randomly sampled from the dataset are shown in Figure 5. Color corresponds to the “fitness” of a molecule for TTF applications: red indicates a better fit, blue, a worse fit. This means that leads are shown in red.

Figure 5 shows that the distribution of red, blue, and intermediate points is far from uniform in the latent space: leads and generally molecules with higher fitness tend to cluster together. Therefore, if we choose a random linear combination of latent space vectors for three red points (leads), it will have an increased chance to end up in the close vicinity of another red point (especially when all leads are sampled from the same cluster). This demonstrates that the JT-VAE model in seeded sampling mode allows to discover most of the leads in the chemical space with fewer iterations than other approaches. We note that there are in total only 56 leads in the validation area of the chemical space comprising 170, 716 molecules, and JT-VAE was capable of discovering 11, i.e,. ≈20 % of those leads in just the first 3,500 unique samples (out of 10, 000 total first samples).

As discussed above, we have analyzed the models’ performance in a relatively small region of chemical space with up to 4 rings without side chains, a region with less than 0.5 million molecules in total. This has allowed us to assess the potential of the models to extrapolate beyond the training set and discover leads in the entire constrained chemical space. In what follows, we apply our conclusions to the discovery of TTF candidates in much larger chemical spaces that cannot be sampled exhaustively.

### 3.4 Filtering Based on Predicting Excitation Energies

Table 2 shows that in our restricted subset of the chemical space, the seeded JT-VAE model generates leads at a rate of (11 + 16)/(559, +, 558) ≈ 2.4%. Though one could apply quantum chemistry methods to all generated molecules to discover the leads, there is a more computationally efficient alternative. We have trained the JT-E network as discussed in Section 2.6 to predict excitation energies for molecules supplied as SMILES strings from the generator. Calculations of excitation energies for the training dataset were done using PM3 (Stewart, 1989), the same SE method as we have used above. Mean absolute errors for the excitation energies for validation set are 0.104, 0.054, and 0.086 eV for the *S*_1_, *T*_1_, and *T*_2_ energies respectively. Note that this is a remarkable accuracy, comparable to the accuracy of the PM3 method itself. The JT-E model is trained independently on the same dataset as the JT-VAE model (see Supplementary Material for details). Based on predicted energies, we have filtered generated molecules according to the same criteria from (Eq. 2): *δ*_*a*_ > 2.3 *eV*, *δ*_*b*_ = *δ*_*c*_ >—0.4 *eV*. Those are looser criteria than for lead selection since we wanted to give a very safe margin of error for the JT-E model (exceeding 2*σ*). Geometries of the molecules satisfying these criteria are then optimized, and excitation energies are computed using PM3. In the next section, we show and discuss the overall workflow for TTF molecules discovery.

### 3.5 Workflow for TTF Molecules Discovery

In order to promote the discovery of real TTF materials, we assembled a multi-step workflow acting in the space of *π*—conjugated compounds. The training datasets consisted of previously used core structures decorated with side groups. We used two types of side groups: tert-butyl and mesityl moieties, as a model of different bulky, but chemically inert substituents that are often used to prevent flat cores from aggregation. We also replaced all amine hydrogens with phenyls, as required to ensure operational stability of the material. The size of the chemical space for compounds with side groups is much larger than for cores only. Therefore the datasets were not exhaustive and included ≈450,000 molecules with positions and types of the side groups selected at random.

**FIGURE 6:**
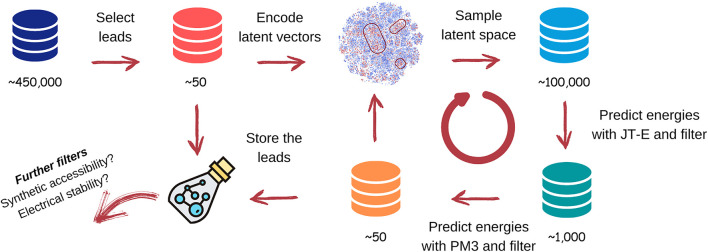
Workflow of the TTF materials discovery. See text for details.

We have investigated three different chemical spaces corresponding to cores: decorated with none, one, and two side chains. We had tried to train a single network on the entire dataset, but our experiments showed that training separate neural networks for each number of side chains allows to increase reconstruction accuracy and accuracy of energies prediction, as well as to achieve better clustering of leads of each type in the chemical space, so we have chosen this strategy. For all molecules from generated chemical spaces, we optimized their geometries and calculated *S*_1_, *T*_1_, and *T*_2_ excitation energies using the SE PM3 approach. We have excluded molecules with unreasonably low energies from each dataset, using criteria outlined in (Eq. 3). The datasets were used to train three JT-VAE generative models and three JT-E energy predicting models (see Supplementary Material for additional data on the architecture and accuracy of JT-VAE and JT-E models). We have selected molecules most suitable for TTF leads from the datasets and utilized them in seeded sampling of the latent space of the autoencoders, using procedures discussed in Section 3.2. The overall discovery workflow for TTF materials is shown in Figure 6.

We have found 75, 58, and 55 leads directly in the dataset with 0, 1, and 2 side chains respectively. The leads were used to seed the generator of JT-VAEs. During each discovery loop, we generated another 100, 000 samples and predicted excitation energies for them using the JT-E network. The molecules satisfying criteria discussed in Section 3.4 were selected for quantum chemical calculation with PM3. Results obtained for each iteration of the workflow cycle are summarized in Table 3.

**TABLE 3:** Gradual increase of the discovered leads for structures with 0, 1, and 2 side chains.

Iteration	Unique after the JT-E filter side chains			Unique after the PM3 filter side chains			Total discovered
	0	1	2	0	1	2	—
0	—	—	—	75	58	55	188
1	357	1,248	1,446	55	195	236	674
2	342	1,378	1,582	67	420	402	1,070

Table 3 shows that all three datasets with nearly 1.5 million molecules initially contained only 188 leads. However, each iteration of the discovery workflow brings ≈400 more new leads. After just two iterations, we have obtained more than a thousand compounds that appear promising for deep blue TTF applications. SMILES notation for those molecules along with calculated PM3 energies are provided in Supplementary Material. Although one can easily continue the discovery cycles, we stopped at the current stage since more than 1,000 leads is already a substantial amount that is not easy to verify experimentally.

## 4 Discussion

In this work, we have presented a computational approach for the discovery of TTF materials, choosing the best deep generative model on the basis of comprehensive experiments with smaller molecules, extending the results onto a much larger chemical space, and producing hundreds of promising leads for new TTF materials. Let us address several points regarding the applied computational methodology. First, a key element of the present study is the use of a very fast approach to quantum chemistry, based on the PM3 method that was not originally designed for the calculation of excited states. Good accuracy was achieved mainly due to additional empirical scaling of the excitation energies. We do not claim that the found scaling factors can be transferred to other applications or have a general scientific value, their application area is presumably limited to fused heterocyclic chromophores. Second, considering the tradeoff between computational cost and accuracy, our PM3-based method obviously represents one of the cheapest and fastest approaches. This was a necessary requirement to perform calculations for hundreds of thousands of compounds within acceptable computational resources, and these calculations were necessary as large datasets were crucial for the successful training of ML-based models. Accuracy of our calculations and the overall high quality of the approach have been validated in a comparison with reliable reference experimental data, both for the excited states energy levels and registered TTF activity. We believe that the resulting list of PubChem hits can be considered as a standalone contribution to the community, providing candidate compounds for blue OLED materials or, at the very least, promising patterns for further research.

One of the possible drawbacks of the current approach lies in the combinatorial nature of the search for all possible valid molecular structures, regardless of their stability or possible synthesizability. This issue can be resolved if we collect only those cores that can be found in PubChem database. This solution is simple and robust, although a lot of novel promising compounds are thus disregarded. In our workflow with ML-based models, we do not impose any additional constraints or filters to disregard unrealistic structures. We prefer to train the models on the complete chemical space, so the predictions are expected to be also correct for synthesizable compounds. After producing the leads, we can decide which molecules to pick up for experimental trials using expert knowledge and other external considerations.

We have demonstrated that ML methods can be applied for successful generation of novel compounds beyond those in the training set. We have been able to provide ML models with large training datasets obtained using SE methods and, at the same time, use ML inference to cover much larger regions of the chemical space. The number of molecules grows rapidly with increasing size of the molecule and heteroatom population. This means that direct calculation of excitation energies is required only for a very small portion of the target space. After that, the training procedure is performed on this dataset, and energies for any other molecule in the chemical space can be inferred from the model in a batch-wise manner with high computational efficiency.

## 5 Conclusion

Using the workflow described above and shown in Figure 6, we have been able to discover hundreds of TTF candidate molecules with *S*_1_, *T*_1_, and *T*_2_ energy levels suitable for TTF application in blue OLED devices. These candidates include more than a dozen of PubChem compounds. After a thorough examination of the suggested leads by experimental chemists, several most promising candidates have been selected for experimental verification. The selection procedures included not only criteria on excitation energies, but also expert assessment of chemical and electrical stability of compounds, their synthetic accessibility, and other considerations. The experimental verification is currently in progress, and we are looking forward to report the results in the nearest future.

We note that the presented approach is not limited solely to TTF molecules, and with reasonable modifications can be applied to other compounds relevant for organic optoelectronic materials.

## Data Availability

The datasets and code for this study can be provided upon reasonable request to the authors.

## Supplementary Material

Data Sheet 1 101 hit without side groups after 3 iterations.

Data Sheet 2 485 hits with 1 side group after 3 iterations.

Data Sheet 3 484 hits with 2 side groups after 3 iterations.

Data Sheet 4 107 PubChem hits.

Data Sheet 5 Supplementary Material.
