# Supplementary material for: Computational Discovery of TTF Molecules with Deep Generative Models
Source: Front Chem. 2021 Dec 23;9:800133. doi: 10.3389/fchem.2021.800133 (PMC8733737; doi:10.3389/fchem.2021.800133)
Supplement: Supplementary file 3 [file DataSheet5.pdf]

Supplementary Material

1 ACCURACY OF NEURAL NETWORKS

1.1 Training and validation data sets

The dataset used for evaluation of performance of generative models (see Section 3.2) consists of $341{,}433$ molecules. The dataset was randomly split in two of the equal size: training and validation data sets, see Fig S1. There is no noticeable difference in distribution of the excitation energies between two data sets, and one can conclude that both data sets equally cover the selected region of the chemical space. Therefore, the observed results of ML models performance should be insensitive to any random shuffles of molecules between the data sets (i.e. insensitive to the particular random splitting).

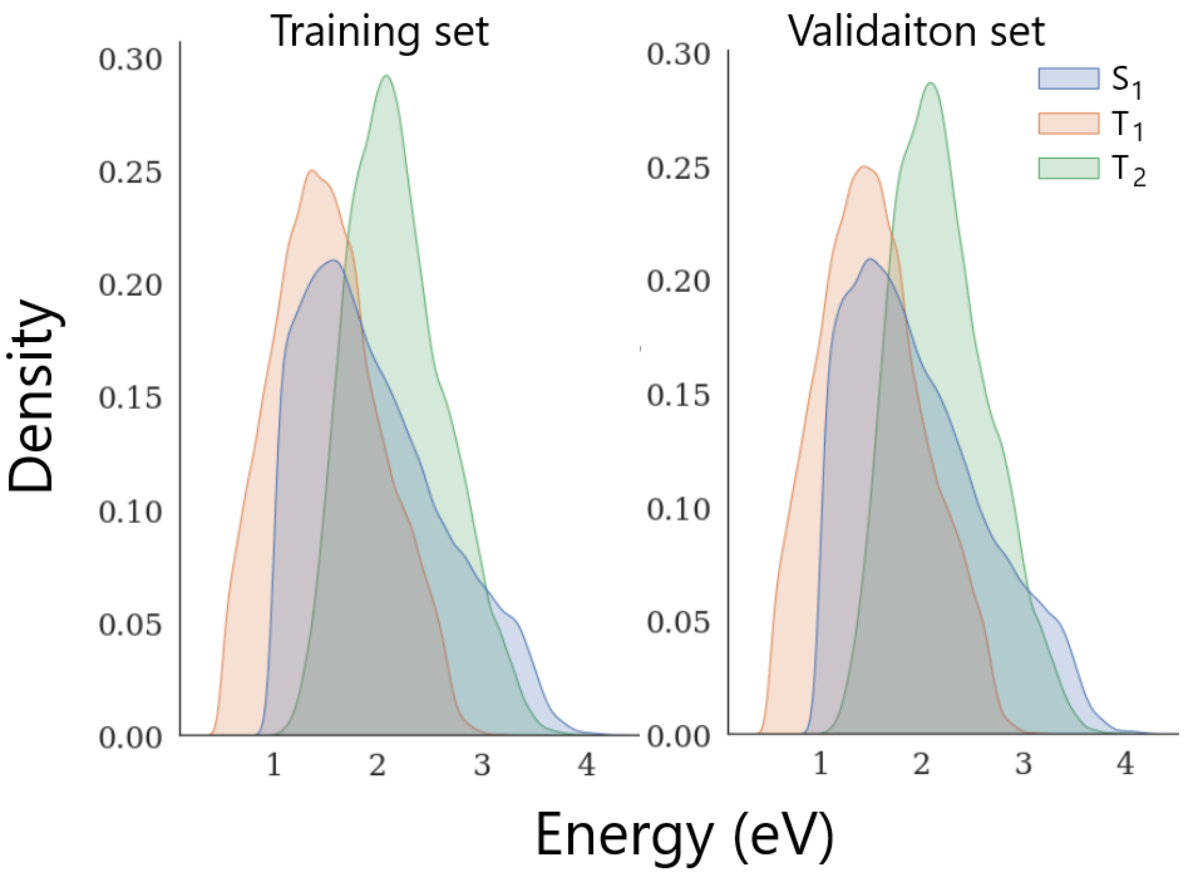

Figure S1. Distributions of S_1 (blue), T_1 (orange) and T_2 (green) energies for the training (left) and validation (right) data sets, which were used for evaluation of performance of different ML models.

1.2 The JT-VAE generative neural network

The JT-VAE neural network was constructed with dimensionality of hidden and latent spaces equal to 450 and 56 respectively. Depth of the graphs and the trees was chosen to be 3 and 20 respectively. To ensure better performance of the autoencoder, the first 10000 steps (warmup) were done without the Kullback-Leibler divergence loss. One of the most important parameters of the autoencoder it its

reconstruction accuracy. Reconstruction accuracy as a function of the training timestep is presented in Fig. S2 for all three networks, corresponding to TTF cores with zero, one and two side chains.

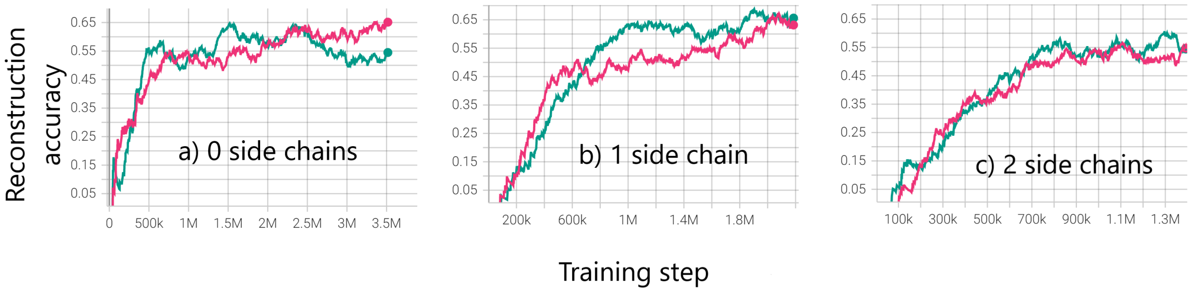

Figure S2. Reconstruction accuracy for JT-VAE NNs. Results for networks trained on datasets with 0, 1, and 2 side chains are shown in graphs **a)**, **b)** and **c)**, respectively. Results for training and validation sets are shown respectively in red and green.

Figure S2 shows that for all datasets, the reconstruction accuracy is close to 60-65%, with the exception for 2 side chains, for which the accuracy is about 5 percentage points lower. Overall, the values correspond quite well to the numbers on reconstruction accuracy reported in the original work on the Junction-Tree VAE (Jin et al., 2018).

1.3 The JT-E model for energies prediction

Out of sample accuracy validation for three JT-E models trained on the sets with 0, 1 and 2 side chains are presented in Table S1. It shows that the highest accuracy is achieved for the dataset with no side chains. Datasets with 1 and 2 side chains feature higher chemical diversity, which makes predictions more difficult. As a result, the accuracy decreases by $\approx 50\%$ for molecules with 2 side chains compared to those with no side chains. It turns out that predictions of singlet excitations are more difficult than for triplet ones. Nevertheless, one can conclude that the prediction performance of JT-E NN is very good, nearly comparable to the accuracy of the PM3 method, which can be measured on the order of ≈ 0.05 eV for the molecules in question (see the discussion in Section 2.1).

2 SCREENING OF THE DATASET

2.1 Benchmarking the methods for computing excitation energies

Results of the validation are presented in Fig. S4. The linear fit used for bias correction is shown with dotted lines. Compounds used in the validation are present in three traits: pure hydrocarbons, nitrogen- and oxygen-containing molecules. These groups are scattered along similar trends in Fig. S4 and were used in statistical analysis without distinction. Fig. S4 also illustrates a surprisingly poor performance of multireference approach. While triplet energies are reasonably good, the prediction of singlet transitions fails entirely. We attribute this issue to larger active spaces required to describe singlet excited states.

	0 side chains			1 side chain			2 side chains		
	S_1	T_1	T_2	S_1	T_1	T_2	S_1	T_1	T_2
MAE	0.104	0.054	0.086	0.139	0.079	0.117	0.155	0.098	0.123
RMSD	0.145	0.077	0.115	0.188	0.108	0.153	0.217	0.136	0.166

Table S1. Mean absolute and root-mean squared errors for JT-E models trained on datasets each comprising $\sim$ 450,000 molecules with 0, 1 and 2 side chains.

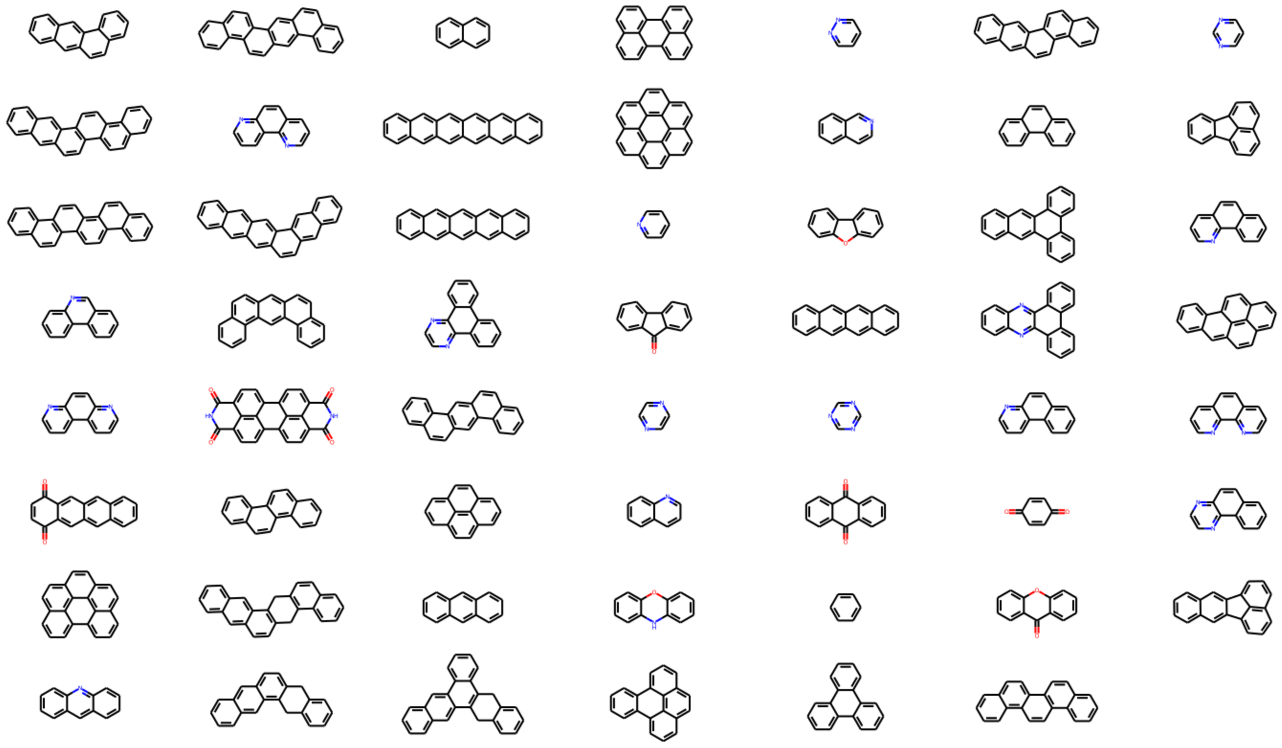

Figure S3. Compounds constituting the validation dataset with known experimental S_1 or T_1 excitation energies (referenced in Section 2.3 of the main text). Nitrogen atoms are highlighted with blue, oxygen atoms with red color.

Although multireference calculations with the (12,12) active space are feasible for molecules of moderate size, blind screening without manual inspection of every particular case seems to be impossible.

Actual values of the thresholds δ_a, δ_b and δ_c are affected by possible inaccuracies in the calculation of energy levels. To find a reasonable estimate of δ_c, we can validate the predictions of corrected PM3 on the experimental dataset (see Fig. S5). It can be seen that deviations are distributed non-uniformly, with larger errors for larger values of δ_c. The worst results are for the six compounds with only one aromatic ring (shown in red on Figure S5). In our search, we have focused on (presumably) larger molecules and lower values of δ_c, so we should be concerned with only the left part of the plot in Fig. S5, demonstrating a standard deviation of about 0.4 eV.

Unfortunately, the same approach cannot be applied to find δ_b, since experimental values of the second triplet energy level are unavailable. The possible error margin here is relatively high because we use empirical factors obtained for T_1 to correct PM3 results for T_2. To define constraints limiting the relevant area of chemical space, we suggest to employ a more qualitative approach. Fig. S6 presents core fragments of compounds with registered TTF activity (Wang et al., 2020), plotted in accordance with their energy losses. For a selected few compounds, external quantum efficiency of the device was reported to exceed statistical limit, thus indicating a favorable alignment of T_1 and T_2 energy levels. These compounds are rubrene (Cheng et al., 2010) (RUB) and perylene (Hoseinkhani et al., 2015) (PER) showed with red markers in Fig. S6. We also added tetracene (TET, square marker) to this group, since the required alignment of T_1 and T_2 in this case is suggested by independent experimental evidence (Völcker et al., 1989; Komfort et al.,

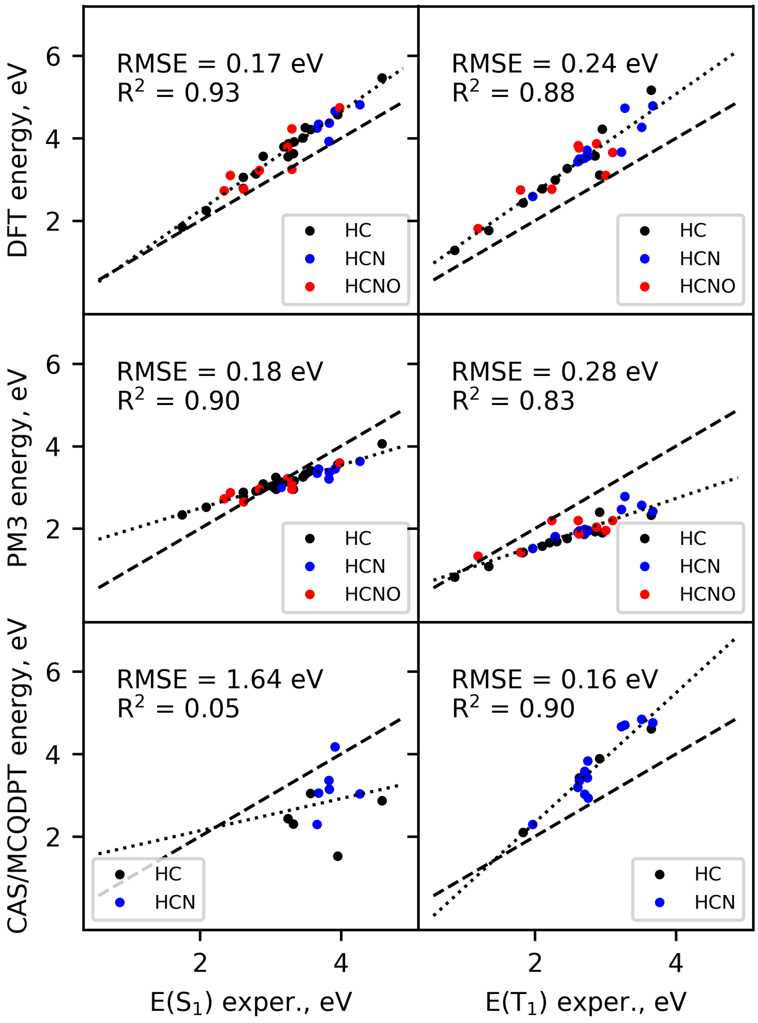

Figure S4. Validation results for energy prediction models; linear fit is shown with dotted lines.

1990; Fallon et al., 2020). It can be seen that the assumption $\delta_b = \delta_c = 0$ fails completely, leaving aside the majority of black points and all red ones. Introduction of a tighter criterion $\delta_c = -0.3\,\text{eV}$ does not change the situation, while allowing some room for statistical errors in calculations. The adequacy of the TTF search relies solely on the δ_b. For $\delta_b = -0.8\,\text{eV}$ almost all points are included in the target area, while lower values ignore many compounds, introducing an obvious error in the important case of perylene.

2.2 Details of the screening

The first generation of the skeleton frames consisted of one 5- and one 6-membered rings. After consecutively applying steps I–III of the structure generation algorithm three times in a row, we obtained all possible frames with at most 4 rings. Step IV produced all possible core compounds within the constraints listed in Section 2.2 of the main text. The corresponding region of the chemical space contains 472505 non-equivalent structures. The size of the subspace is sufficiently small to be treatable with SE methods of quantum chemistry, but at the same time large enough to be subjected to the search of promising TTF candidates and to be used for the development and validation of ML models. After that, we conducted geometry optimization using PM3. On the optimized structures, three first singlet and triplet excitation energies were calculated in *Gaussian 16* at the configuration interactions singles (CIS) level using PM3 Hamiltonian. For 10035 structures (about 2% of the total amount) simulations failed, primarily due to the

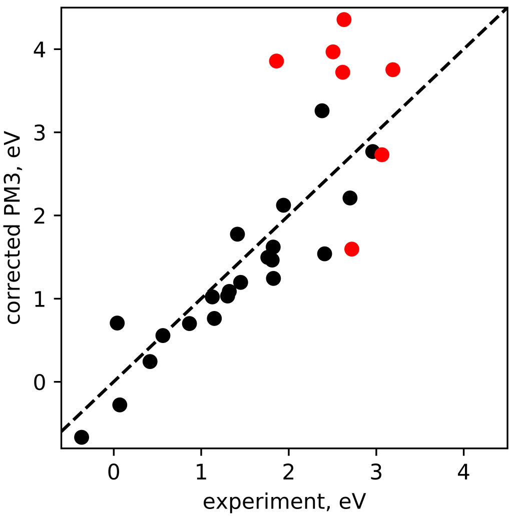

Figure S5. Comparison of experimental and calculated values of $\delta_c = 2T_1 - S_1$. Red markers denote compounds consisted of one ring. Dashed line corresponds to exact coincidence.

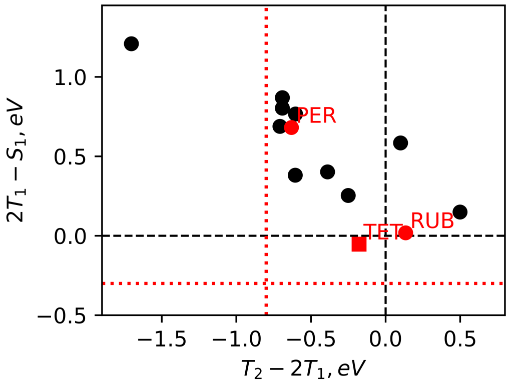

Figure S6. Calculated energy levels of existing TTF compounds. Cases with experimental evidences of prohibited triplet decay channel are colored with red. Black dashed lines correspond to ideal TTF criteria: $\delta_b = \delta_c = 0$. Red dotted lines correspond to the TTF criteria applied in actual calculations.

unconverged optimization procedure. After application of TTF criteria, the majority of core compounds were fileterd out, leaving only 5690 candidates.

Table S2: Candidates for the TTF core compounds found in *PubChem* database.

ID	CID	SMILES	FIGURE	$E(S_0)$, eV	$E(T_1)$, eV	$E(T_2)$, eV
Group ancestor: anthracene						
1	192719	c1cnc2cc3cccnc3cc2c1	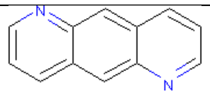	3.39	2.04	3.36

2	12867025	c1ccc2cc3cnncc3cc2c1	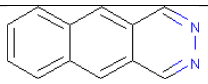	3.01	2.01	3.26
3	15115274	c1cnc2cc3cnccc3cc2c1	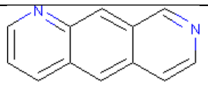	3.38	2.01	3.25
4	19041920	c1ncc2cc3ncncc3cc2n1	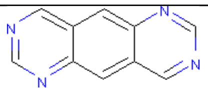	3.07	2.25	3.73
5	89231701	c1cnc2cc3ncncc3cc2c1	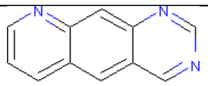	3.11	2.15	3.52
6	69173896	c1cnc2cc3ccncc3cc2c1	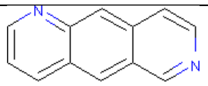	3.38	2.01	3.27
7	520238	c1ccc2cc3ncccc3cc2c1	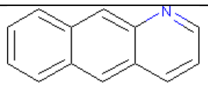	3.31	1.91	3.17
8	13064692	c1cc2cc3cnccc3cc2cn1	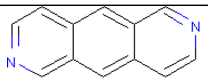	3.35	1.97	3.18
9	22999777	c1cc2cc3ccncc3cc2cn1	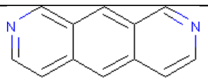	3.40	1.99	3.18
10	601692	c1ccc2cc3cnccc3cc2c1	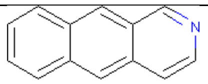	3.29	1.89	3.09
11	22745371	c1ccc2cc3ncncc3cc2c1	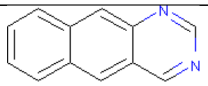	3.13	2.01	3.33
12	12886698	c1ccc2cc3nccnc3cc2c1	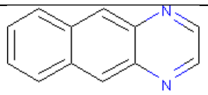	2.85	2.01	3.34
13	2750235	c1cnc2cc3ncccc3cc2c1	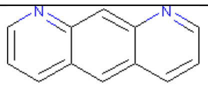	3.40	2.04	3.34
14	19886268	c1cnc2cc3cncnc3cc2c1	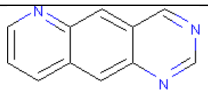	3.13	2.15	3.52

15	8418	c1ccc2cc3ccccc3cc2c1	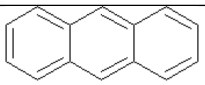	3.23	1.81	3.01
16	21050939	c1cnc2cc3nccnc3cc2c1	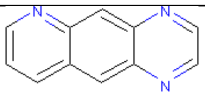	2.85	2.14	3.50

Group ancestor: tetracene

17	59467607	c1cnc2cc3cc4ncccc4cc3cc2c1	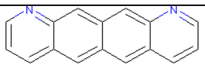	2.70	1.42	2.58
18	13287587	c1ccc2cc3cc4cnccc4cc3cc2c1	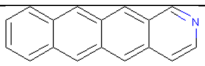	2.61	1.32	2.41
19	59467675	c1cnc2cc3nc4ccccc4cc3cc2c1	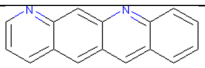	2.68	1.45	2.48
20	59467659	c1nncc2cc3cc4cnncc4cc3cc12	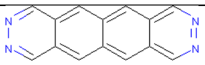	2.84	1.58	2.76
21	71359095	c1ccc2cc3cc4ncncc4cc3cc2c1	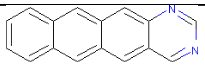	2.66	1.40	2.55
22	88456039	c1ccc2nc3cc4cnccc4cc3cc2c1	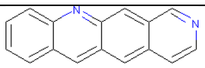	2.68	1.44	2.45
23	59467711	c1ccc2cc3cc4nccnc4cc3cc2c1	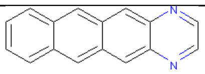	2.66	1.40	2.55
24	102403997	c1cnc2cc3nc4cccnc4cc3nc2c1	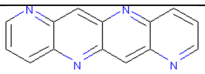	2.74	1.69	2.73
25	88455570	c1ccc2nc3cc4ccncc4cc3cc2c1	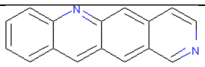	2.67	1.44	2.45
26	12313103	c1ccc2cc3cc4ncccc4cc3cc2c1	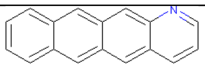	2.62	1.33	2.44
27	59422764	c1cnc2cc3cc4nccnc4cc3cc2n1	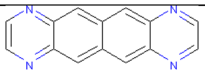	2.74	1.58	2.83
28	59467628	c1cnc2cc3cc4ccccc4nc3cc2c1	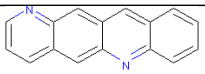	2.69	1.45	2.48
29	12586670	c1ccc2cc3cc4cnncc4cc3cc2c1	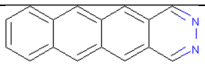	2.68	1.41	2.53

30	59467676	c1cnc2cc3cc4cccnc 4cc3cc2c1	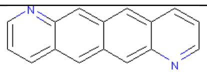	2.70	1.42	2.58
31	59467705	c1cc2cc3cc4ccncc4 cc3cc2cn1	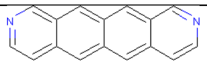	2.69	1.39	2.49
32	59467631	c1cc2cc3cc4cnccc4 cc3cc2cn1	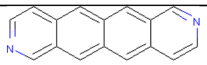	2.67	1.38	2.49
33	137459010	c1ccc2nc3cc4nccnc 4cc3cc2c1	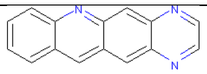	2.72	1.53	2.58
34	12309611	c1ccc2nc3cc4ccccc 4nc3cc2c1	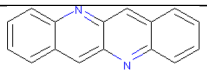	2.67	1.49	2.41
35	130290887	c1ccc2cc3nc4cccnc 4cc3cc2c1	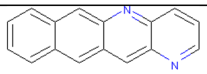	2.66	1.45	2.54
Group ancestor: isobenzofurane						
36	45080545	c1ncc2nocc2n1	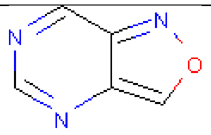	2.86	2.12	3.60
37	54059110	c1cc2conc2cn1	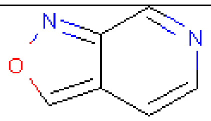	3.33	1.89	3.49
38	12355694	c1cc2cocc2cn1	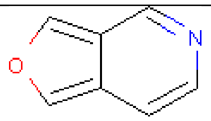	3.65	1.79	3.42
39	18465322	c1ncc2cocc2n1	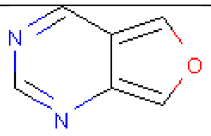	3.15	2.00	3.82
40	56972324	c1cc2nocc2cn1	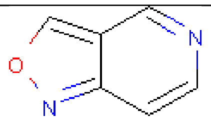	3.52	2.02	3.51
41	121361673	c1nncc2cocc12	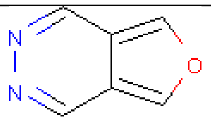	3.01	2.02	3.70
42	18442745	c1cnc2cocc2c1	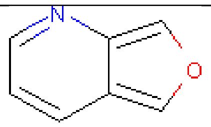	3.63	1.81	3.50
43	22574589	c1cnc2conc2c1	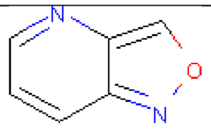	3.45	1.97	3.54

44	15254832	c1cnc2nocc2c1	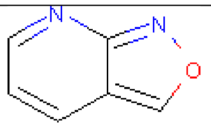	3.19	2.02	3.60
45	67498	c1ccc2nocc2c1	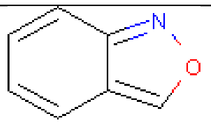	3.39	1.78	3.41
46	23146843	c1cnc2cocc2n1	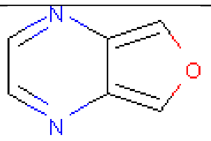	2.87	1.98	3.51

Group ancestor: pyrene

47	10465507	c1cc2cncc3ccc4cncc1c4c23	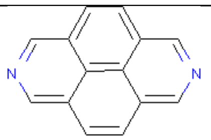	3.30	2.02	3.36
48	9132	c1cc2ccc3cncc4ccc(c1)c2c34	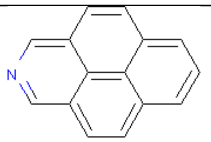	3.30	2.03	3.37
49	31423	c1cc2ccc3cccc4ccc(c1)c2c34	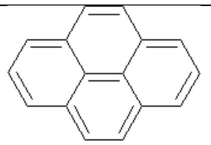	3.27	2.04	3.38

Group ancestor: furane

50	118210311	c1noc2cnoc12	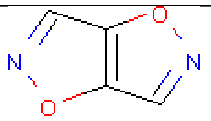	3.72	2.43	4.20
51	45122577	c1cc2cc3c[nH]cc3cc2o1	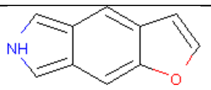	3.30	1.78	2.85
52	45120275	c1nc2c(ccc3cocc32)o1	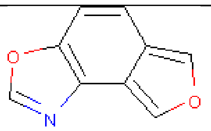	3.29	1.70	3.08
53	53471472	c1cc2cn[nH]c2c2nocc12	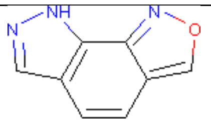	3.34	2.04	3.38
54	45120272	c1nc2ccc3cocc3c2o1	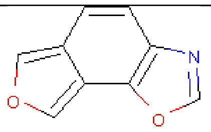	3.42	1.72	3.21

55	136344757	c1cc2nocc2[nH]1	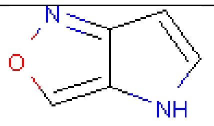	3.11	1.87	3.05
56	70281749	c1ccc2c(c1)[nH]c1c3cocc3ccc21	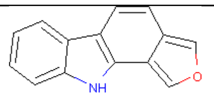	3.08	1.71	2.85
57	141022627	c1ccc2c(c1)[nH]c1cc3cocc3cc12	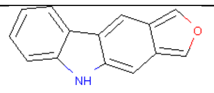	2.68	1.38	2.74
58	15866930	c1ccc2c(c1)cn1c3cccc3oc21	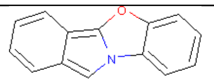	3.19	1.80	2.92
59	57357167	c1cc2oncc2c2cocc12	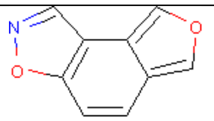	3.23	1.73	3.05
60	45120274	c1nc2cc3cocc3cc2o1	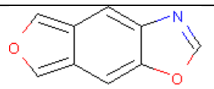	3.18	1.45	2.98
61	13287595	c1ccc2cc3cc4occc4cc3cc2c1	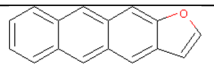	2.92	1.60	2.57
62	55288078	c1cc2cocc2[nH]1	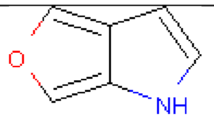	3.67	1.83	3.05
63	22599150	c1ccc2cc3cc4ocnc4cc3cc2c1	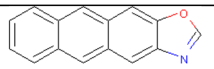	2.98	1.67	2.77
64	20093437	c1nc2cocc2[nH]1	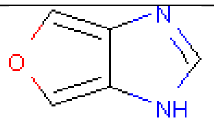	3.93	2.08	3.44
65	136192814	c1cc2conc2[nH]1	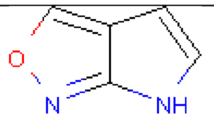	3.31	2.00	3.23
66	129857652	c1cnc2ccc3nc4cocc4cc3c2c1	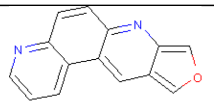	2.77	1.34	2.61
67	45087483	c1cc2coc3ccc1n23	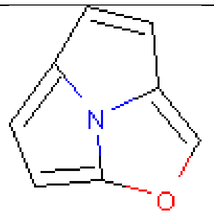	2.74	1.82	2.92

68	129740670	c1ccc2c(c1)ccc1nc3cocc3cc12	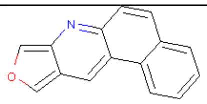	2.79	1.34	2.57
69	15350289	c1cc2c3cocc3ccc2c2cocc12	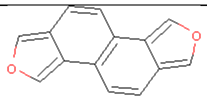	2.83	1.42	2.08
70	45120728	c1nc2c(cnc3cocc32)[nH]1	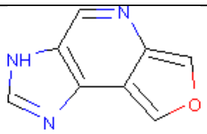	3.55	2.08	3.38
71	57357844	c1occ2cc3oncc3cc12	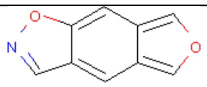	3.11	1.44	2.99
72	14322637	c1cc2ccc3coc4ccc(c1)c2c34	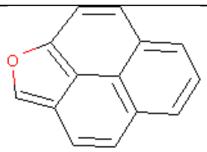	3.16	1.69	3.05
73	45078668	c1nc2c(ccc3cocc32)[nH]1	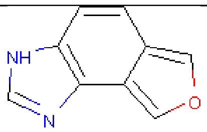	3.42	1.89	3.21
74	3540653	c1nc2c(ccc3nocc32)o1	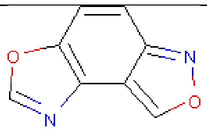	3.19	1.81	3.19
75	57352075	c1cc2c(ccc3conc32)o1	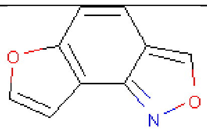	3.14	1.81	2.96
76	132204277	c1cc2ccc3cocc3c2c2cocc12	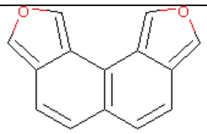	2.94	1.43	2.08
77	22599148	c1ccc2cc3c(ccc4ncoc43)cc2c1	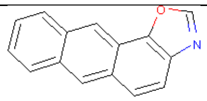	3.10	1.85	2.95

Group ancestor: others

78	21887470	c1ccc2c(c1)nc1cnc3coccc3c12	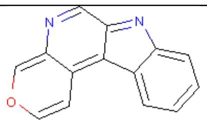	2.73	1.38	2.24
79	57116324	c1ccc2c(c1)[nH]c1cc3c[nH]cc3cc12	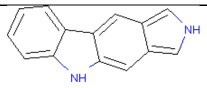	2.89	1.78	2.85
80	129732091	C1=C2ONC=C2Oc2ccccc21	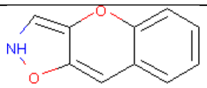	2.87	1.63	2.62
81	13287594	c1ccc2cc3c[nH]cc3cc2c1	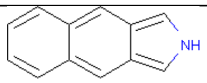	2.75	1.31	2.63
82	45121711	c1ccc2nc3c[nH]cc3nc2c1	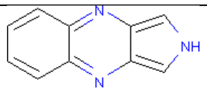	2.70	1.53	2.71
83	12366601	c1ccc2cn3ncnc3cc2c1	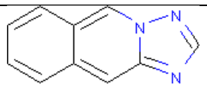	2.78	1.71	3.29
84	13764187	c1cc2c[nH]cc2cn1	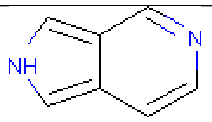	3.90	2.23	3.67
85	129652737	C1=COC2=CNOC2=C1	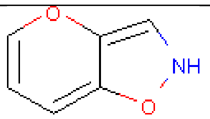	2.70	1.45	2.39
86	54455968	c1ccc2nc3ncnn3cc2c1	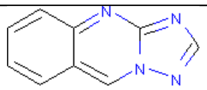	2.67	1.84	3.34
87	66579102	C1=C2NNC=C2Oc2ccccc21	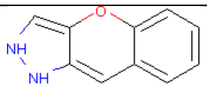	2.95	1.60	2.62
88	129826334	C1=CC2=CNNC2=CO1	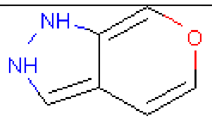	3.08	1.55	2.31
89	139524815	C1=Cc2c([nH]c3ccccc23)NN1	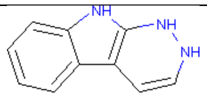	2.69	1.81	2.98
90	3013853	c1ccc2c[nH]cc2c1	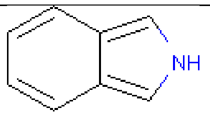	3.81	2.08	3.57

91	90729303	c1cc2c[nH]c3ccc4[nH]cc1c4c23	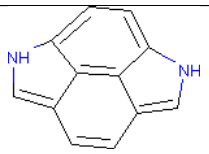	3.19	1.81	3.09
92	53639421	c1ccc2c(c1)cn1c3c cccc3[nH]c21	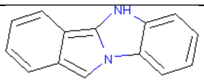	2.97	1.81	2.87
93	129883915	C1=C2Oc3ccccc 3C=C2Oc2cncnc21	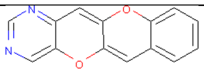	2.74	1.50	2.43
94	129790303	C1=COC2=CC=NO C2=C1	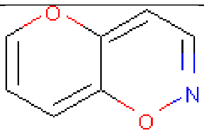	2.74	1.30	2.17
95	54151620	c1ccc2c(c1)cn1c3n cncc3[nH]c21	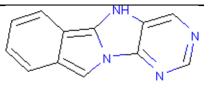	2.67	1.66	2.87
96	426233	C1=CNNC=C1	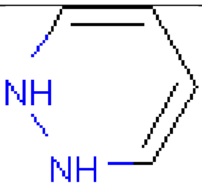	2.62	1.77	3.65
97	129737116	c1ccc2c(c1)cn1[nH]ccc21	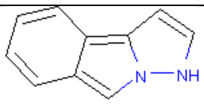	2.89	1.67	2.86
98	70213721	C1=COC2=Cc3cc ccc3OC2=C1	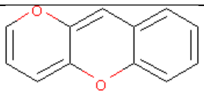	2.83	1.32	2.17
99	136347748	c1cc2c3c([nH]cc3c 1)N=N2	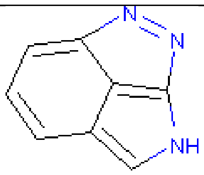	2.69	1.89	3.07
100	88625285	c1ccc2c(c1)ccc1cc 3c[nH]cc3cc12	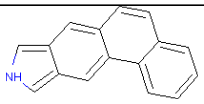	2.91	1.60	2.69
101	70043787	C1=NOC2=Cc3cc ccc3OC2=C1	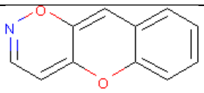	2.97	1.46	2.44

102	129782488	c1ccc2c(c1)cc1ccc c3ncoc2c13	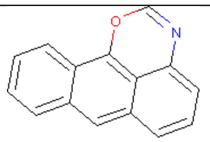	2.81	1.57	2.58
103	69101505	C1=COC2=CNNC2 =C1	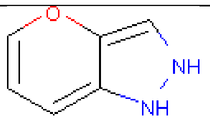	2.82	1.42	2.39
104	13764185	c1cnc2c[nH]cc2c1	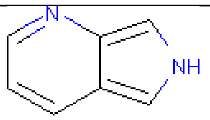	3.85	2.25	3.77
105	17860321	C1=CNNC=N1	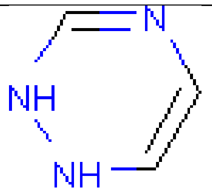	2.60	1.98	4.00
106	70552035	c1ccc2c(c1)cn1[nH]cnc21	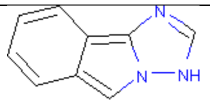	2.73	1.64	3.08
107	22714981	c1ccc2nc3c[nH]cc3 cc2c1	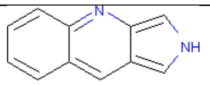	2.75	1.43	2.68

REFERENCES

Cheng, Y. Y., Fückel, B., Khoury, T., Clady, R. G., Tayebjee, M. J., Ekins-Daukes, N., et al. (2010). Kinetic analysis of photochemical upconversion by triplet-triplet annihilation: beyond any spin statistical limit. *J. Phys. Chem. Lett.* 1, 1795–1799

Fallon, K. J., Churchill, E. M., Sanders, S. N., Shee, J., Weber, J. L., Meir, R., et al. (2020). Molecular engineering of chromophores to enable triplet–triplet annihilation upconversion. *Journal of the American Chemical Society* 142, 19917–19925

Hoseinkhani, S., Tubino, R., Meinardi, F., and Monguzzi, A. (2015). Achieving the photon up-conversion thermodynamic yield upper limit by sensitized triplet–triplet annihilation. *Phys. Chem. Chem. Phys.* 17, 4020–4024

Jin, W., Barzilay, R., and Jaakkola, T. (2018). Junction tree variational autoencoder for molecular graph generation. In *International Conference on Machine Learning* (PMLR), 2323–2332

Komfort, M., Löhmannsröben, H.-G., and Salthammer, T. (1990). The temperature dependence of photophysical processes in perylene, tetracene and some of their derivatives. *Journal of Photochemistry and Photobiology A: Chemistry* 51, 215–227

Völcker, A., Adick, H.-J., Schmidt, R., and Brauer, H.-D. (1989). Near-infrared phosphorescence emission of compounds with low-lying triplet states. *Chemical physics letters* 159, 103–108

Wang, X., Tom, R., Liu, X., Congreve, D. N., and Marom, N. (2020). An energetics perspective on why there are so few triplet–triplet annihilation emitters. *J. Mater. Chem. C* 8, 10816–10824
